# Ag Nanoparticle-Decorated Cu_2_S Nanosheets for Surface Enhanced Raman Spectroscopy Detection and Photocatalytic Applications

**DOI:** 10.3390/nano11102508

**Published:** 2021-09-26

**Authors:** Osama Nasr, Jian-Ru Jiang, Wen-Shuo Chuang, Sheng-Wei Lee, Chih-Yen Chen

**Affiliations:** 1 Department of Materials and Optoelectronic Science, National Sun Yat-Sen University, Kaohsiung 804201, Taiwan; osamanasr@nsysu.edu.tw (O.N.); egg03@mail.mirdc.org.tw (W.-S.C.); 2 Institute of Materials Science and Engineering, National Central University, Taoyuan 320317, Taiwan; icq200148@gmail.com

**Keywords:** Cu_2_S nanosheets, Ag nanoparticles, SERS probes, photodegradation, photocatalysts

## Abstract

In this article, we demonstrate a facile, rapid, and practical approach to growing high-quality Cu_2_S nanosheets decorated with Ag nanoparticles (NPs) through the galvanic reduction method. The Ag/Cu_2_S nanosheets were efficiently applied to the surface-enhanced Raman scattering (SERS) and photocatalytic degradation applications. The photodegradation of RhB dye with the Ag/Cu_2_S nanosheets composites occurred at a rate of 2.9 times faster than that observed with the undecorated Cu_2_S nanosheets. Furthermore, the Ag/Cu_2_S nanosheets displayed highly sensitive SERS detection of organic pollutant (R6G) as low as 10^−9^ M. The reproducibility experiments indicated that the Ag/Cu_2_S nanosheets composites could be used for dual functionality in a new generation of outstandingly sensitive SERS probes for detection and stable photocatalysts.

## 1. Introduction

Nanostructured metal chalcogenides have been widely studied for their unique applications because of their small-size effects. A variety of anisotropic nanostructures such as nanoparticles [1], nanowires [2], and nanosheets [3] are usually preferable to bulk materials in technological applications. They have been thoroughly studied as potential building blocks for the fabrication of novel nanodevices. Among various metal chalcogenide semiconductors, copper(I) sulfide (Cu_2_S) has an energy bandgap of 1.21 eV that could be applied in optoelectronics and photocatalysis [4,5,6]. Cu_2_S nanomaterials are quite attractive because many of the proposed fabrication methods are inexpensive and lend themselves well to mass production [4,5,6,7,8]. Cu_2_S nanostructures, including nanocrystals, nanowires, and nanosheets, have aided in developing highly efficient photocatalysts for the environmental photocatalytic degradation of organic pollutants [6,7,8].

Meanwhile, chalcogenide semiconductors combined with noble metals like Au or Ag nanoparticles (NPs) have received massive attention; they have been extensively explored for their potential applications in the fields of surface-enhanced Raman scattering (SERS) [9,10,11,12] and photocatalysis [6,13,14]. The photocatalytic mechanism in noble metals–semiconductor composites has been discussed extensively [15,16]. The Schottky barrier at the interface has been proven to facilitate the charge separation and hence improve the photocatalytic efficiency [17]. On the other hand, SERS has been recognized as a powerful technique for the ultra-sensitive detection of a variety of chemical and biochemical molecules [18,19]. The SERS effect has been primarily attributed to the intensified electric field associated with the local surface plasmon resonance (LSPR) of metallic nanoparticles and electromagnetic (EM) enhancement. Previous studies demonstrated that Ag NPs feature greater advantages over Au and Cu NPs owing to their stronger plasmonic effect and ease of synthesis [20]. The enhancement factor (EF) of the SERS signal can be up to 10^3^–10^14^, enabling single-molecule detection in some cases [21,22,23]. The extremely high enhancement signal has made SERS a very powerful tool to detect trace amounts of molecules adsorbed in the surface of noble metals [24].

Herein, we demonstrate a facile and environmentally friendly approach to synthesize Cu_2_S nanosheets decorated with Ag NPs (Ag/Cu_2_S nanosheets) by using a convenient galvanic reduction method. In the SEM nanostructure investigation, Ag NPs were preferentially deposited on the edge of Cu_2_S nanosheets, where many activated sites led to the easy deposition of Ag NPs. Ordered Ag NPs are extremely attractive catalysts because of their significant catalytic activities [25], size- or shape-dependent optical properties [26], and promising chemical and biological sensing based on SERS and localized surface plasma resonance (LSPR) [16,27,28,29]. The results suggest that Ag/Cu_2_S nanosheets have a high-performance SERS effect that can be applied in optoelectronic devices; furthermore, they show promising photocatalytic potential for new types of future applications.

## 2. Materials and Methods

### 2.1. Preparation of Cu_2_S Nanosheets

In a typical chemical experiment procedure, 8 mmol of sulfur powder (≥99.0%, Sigma-Aldrich, St. Louis, MO, USA), 15 g of sodium hydroxide (NaOH, ≥98%, Sigma), 14 mL of ethylenediamine (≥99%, Sigma), and 80 μL of hydrazine (98%, Sigma) were sequentially dissolved in 80 mL of deionized (DI) water, and reaction temperature was controlled at 50 °C. A cleaned polycrystalline copper foil (Nanya corporation, Taipei, Taiwan) was used as the substrate to grow Cu_2_S nanosheets, and it was placed in the solution for 30 s. The obtained black substrate was taken out and cleaned with isopropyl alcohol and DI water three times and finally stored under a vacuum.

### 2.2. Preparation of Ag/Cu_2_S Nanosheets

Ag-NP-decorated Cu_2_S nanosheets (Ag/Cu_2_S) were prepared via the galvanic reduction method. A 0.01 M silver nitrate (AgNO_3_, Merck, Darmstadt, Germany) solution was prepared and heated at different temperatures (20, 30, and 40 °C). The black Cu_2_S nanosheets were immersed into the AgNO_3_ solution for 10 s and then immediately removed from the solution. The Ag/Cu_2_S nanosheets were thoroughly cleaned with isopropyl alcohol and DI water several times and finally preserved under a nitrogen atmosphere.

### 2.3. Characterizations

Several analytical techniques were used for the morphological characterization of the Cu_2_S and Ag/Cu_2_S nanosheets. A scanning electron microscope (SEM Quanta 200, FEI, Hillsboro, OR, USA) equipped with an energy-dispersive X-ray spectrometer was used to determine the morphologies and chemical composition of the Cu_2_S and Ag/Cu_2_S nanosheets. The high-resolution lattice images of the Cu_2_S nanostructure were obtained using a transmission electron microscope (TEM JEM 2100, JEOL, Tokyo, Japan), operating at 200 keV. X-ray diffraction (XRD D2, Bruker, Karlsruhe, Germany) was used to understand the crystallography of the Cu_2_S and Ag/Cu_2_S nanosheets on the Cu foil and was recorded with a diffractometer using Cu Kα radiation at the rate of 0.02 per step. In order to investigate the process of electron transfer on the Ag/Cu_2_S nanosheets, X-ray photoelectron spectroscopy (XPS, Thermo Scientific, Waltham, MA, USA) was used to analyze the Ag and Cu spectra at the interface between the Ag NP and Cu_2_S nanosheets. A UniRAM-Raman spectrometer (ProTrusTech, Tainan, Taiwan) equipped with a 532 nm laser was used to record the SERS spectra of a rhodamine 6G (R6G, 99%, Merck) probe dropped over the prepared Ag/Cu_2_S substrates. The photocatalytic activity of Ag/Cu_2_S towards rhodamine B (RhB) degradation was evaluated under UV irradiation using a 200W HgXe arc lamp. The photodegradation experiments were performed by using Ag/Cu_2_S films of 1 × 1 cm^2^ area immersed in 6 mL of 10 μM RhB (98%, Sigma) solution. UV-visible absorption spectra of the RhB solution were recorded using a spectrophotometer (Evolution 60S, Thermo Scientific, Waltham, MA, USA).

## 3. Results and discussion

### 3.1. Morphology and Structure of Ag/Cu_2_S Nanosheets

**Figure 1:**
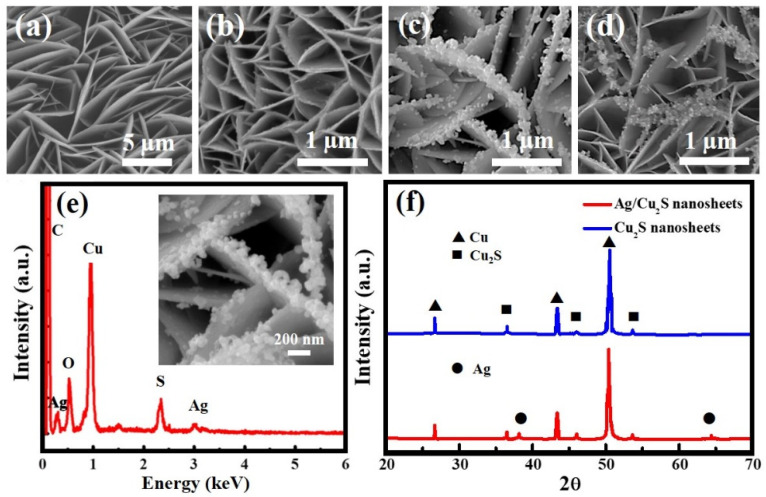
SEM images of (**a**) Cu_2_S nanosheets and Ag/Cu_2_S prepared at (**b**) 20 °C, (**c**) 30 °C, and (**d**) 40 °C. (**e**) EDX analysis of Ag/Cu_2_S nanosheets prepared at 30 °C. The inset SEM image reveals 100 nm Ag NPs were well deposited on the edge of 30 °C immersed Ag/Cu_2_S nanosheets. (**f**) XRD patterns of Cu_2_S nanosheets and Ag/Cu_2_S nanosheets prepared at 30 °C.

Figure 1a shows the SEM image of the nanosheets morphology of the Cu_2_S with an average diameter of around 5 µm and thickness below 20 nm. The transmission-electron-microscopy (TEM) images shown in Figure S1 provide direct crystal information on the Cu_2_S nanosheets. Figure 1b–d shows the SEM images of Ag/Cu_2_S nanosheets grown in AgNO_3_ solution at different temperatures of 20, 30, or 40 °C, respectively; these images indicate that the morphology of Ag NPs is dependent on the temperature of the galvanic AgNO_3_ solution. As shown in Figure 1c, Ag NPs were preferentially located on the edge of the Cu_2_S nanosheets, which could be explained by the localization of the electrons on the edge of the Cu_2_S nanosheets. Lower ion diffusion can result in much smaller Ag clusters at a relatively low reaction temperature that decreases the decorating rate of Ag NPs on the edge of the Cu_2_S nanosheets. In contrast to low temperatures, Ag NPs are prone to be aggregated at relatively high reaction temperatures. After comparing the SEM images of Figure 1a–d to explore the optimal temperature value for fabricating high-quality Ag/Cu_2_S nanosheets, we found it to be 30 °C. The successful decoration of Ag NPs onto the surface of Cu_2_S nanosheets was also confirmed by the EDX elemental analysis as shown in Figure 1e. The inset SEM image of Figure 1e shows the high-quality and uniform Ag/Cu_2_S nanosheets of the existing 100 nm Ag NPs that were well-deposited on the edge of the Cu_2_S nanosheets. Moreover, the crystal structure of the Ag/Cu_2_S nanosheets was determined by X-ray diffraction. Figure 1f shows the typical XRD patterns of the undecorated Cu_2_S nanosheets and Ag/Cu_2_S nanosheets. All diffraction peaks could be ascribed to the orthorhombic crystal of Cu_2_S (JCPDS No. 23-0961) with lattice constants of a = 1.35 nm, b = 2.73 nm, and c = 1.19 nm [8], apart from the three additional peaks (2θ = 27.52°, 43.32°, and 50.45°) corresponding to the Cu foil. Even though the peak intensity of the Ag NPs of the Ag/Cu_2_S nanosheets was lower than that of the Cu_2_S structures in the XRD patterns, the appearance of the peak of the Ag (111) diffraction plane is clear in Figure 1f, indicating that Ag NPs were successfully embedded in the interface of the Cu_2_S nanosheets.

### 3.2. SERS Evaluation of Ag/Cu_2_S Nanosheets

**Figure 2:**
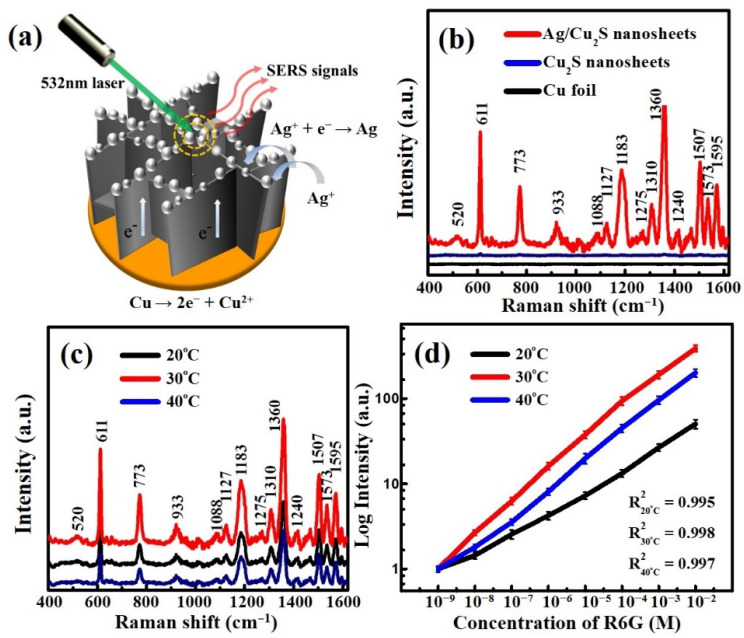
(**a**) Illustration of SERS sensing of Ag/Cu_2_S nanosheets for detection of rhodamine 6G (R6G) dye molecule. (**b**) SERS spectra of R6G dye adsorbed on three different substrates: Cu foil, Cu_2_S, and Ag/Cu_2_S nanosheets. (**c**) SERS spectra of R6G using Ag/Cu_2_S prepared at different temperatures of 20, 30, and 40 °C. (**d**) Linear correlation between the concentration of R6G and the intensity of characteristic SERS peak at different immersed temperatures of 20, 30, and 40 °C.

Raman spectroscopy is a spectroscopic technique to observe vibrational, rotational, and other low-frequency modes in a system. Active SERS substrates can provide excellent sensitivity to measure the vibrational spectra of specific adsorbed molecules [9,10]. However, the intensity and reliability of SERS critically depend on the surface morphology and geometry of the nanostructured materials. The SERS performance of the Ag/Cu_2_S nanosheets was explored using R6G dye as a probe molecule, which is widely used in the field of SERS applications. As shown in Figure 2b, the Ag/Cu_2_S nanosheets obtained at 30 °C possess brilliant SERS performance in contrast to the poor SERS performance of the undecorated Cu_2_S nanosheets. Through four repeated experiments, we provide evidence that the results are reproducible. The reliability tests of SERS spectra, as shown in Figure S2, were collected in a 10^−4^ M R6G dye solution by using repeated Ag/Cu_2_S nanosheets. Comparing data from the four repeated experiments, the results showed a slight shift in the SERS spectra (see Figure S2 in Supplementary Files).

According to Figure 2c, Ag/Cu_2_S nanosheets prepared at 30 °C possess better SERS performance than those obtained at 20 and 40 °C, which can be attributed to the uniform distribution of the Ag NPs on the edge of the Cu_2_S nanosheets at 30 °C. As shown in Figure 2d, SERS intensity was accordingly decreased, while the concentration of the R6G dye molecule decreased. However, SERS signals still detected as low a concentration of the R6G dye molecule at 10^−9^ M, as shown in Figure 2d, which presented a linear experimental relationship between SERS intensity and the concentration of the R6G dye molecule. The correlation refers to the straight-line relationships between 10^−2^ and 10^−9^ M, with a constant of proportionality that suggested that the number of adsorption sites with high Raman enhancement was large enough to accommodate a considerable range of sample concentrations. The results showed that there was a good linear correlation between characteristic SERS peak intensity and R6G concentration with an R^2^ of around 99.5% to 99.8%. 

### 3.3. Photocatalytic Activity of Ag/Cu_2_S Nanosheets

**Figure 3:**
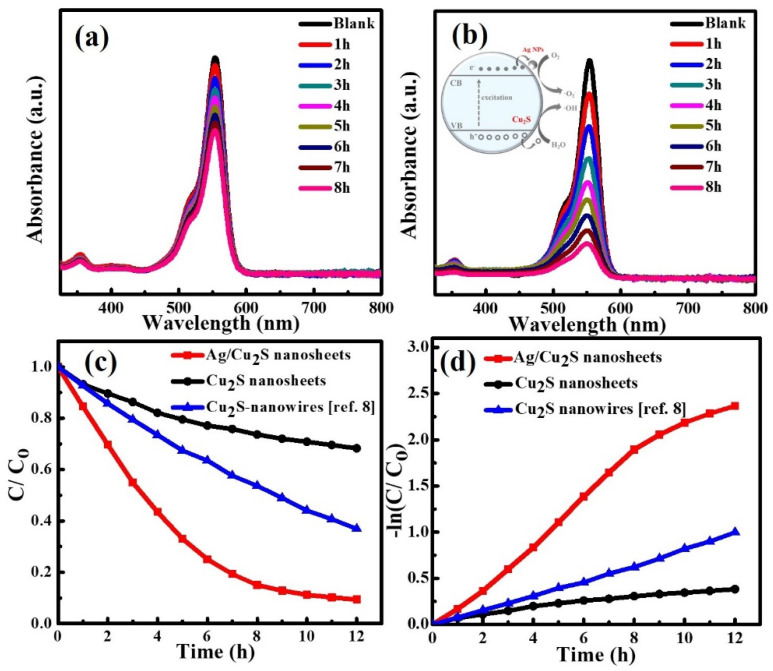
UV-visible absorption of RhB dye during the photodegradation process using (**a**) Cu_2_S and (**b**) Ag/Cu_2_S nanosheets. The inset illustrates the electron transfer process in Ag/Cu_2_S under light irradiation. (**c**) Relative change in concentration of the RhB dye during the photodegradation over different photocatalysts. (**d**) Kinetics of photodegradation and apparent rate constant (K) using three different catalysts. Photodegradation results of Cu_2_S nanowires were mentioned in our previous report [8].

The photocatalytic activity of Cu_2_S and Ag/Cu_2_S nanosheets was investigated using RhB dye, and the process was monitored by UV-visible spectroscopy as shown in Figure 3a,b. A profound decrease in the absorbance of RhB was observed when the Cu_2_S nanosheets were decorated with Ag NPs at 30 °C. The improved photocatalytic activity can be attributed to the enhanced electron-hole (e^−^-h^+^) separation resulting from the Schottky barrier at the interface of the Ag/Cu_2_S nanosheet heterojunction, as illustrated by the inset in Figure 3b [15,16]. Therefore, Ag NPs can act as electron sinks, thereby reducing the recombination of photoinduced electrons and holes, and can prolong the lifetime of the (e^−^-h^+^) pairs. The photodegradation efficiency (η) of the RhB dye molecule was calculated based on the absorbance for a period of 12 h of irradiation divided by the initial absorbance of the pollutant solution. Figure 3c demonstrates that the efficiency (η) of the RhB photodegradation using the Ag/Cu_2_S nanosheets and undecorated Cu_2_S nanosheets was 90.3 and 31.3, respectively. Interestingly, the photocatalytic activity of the Ag/Cu_2_S nanosheets surpassed that of the Cu_2_S nanowires in our previous work [8]. Figure 3d shows that the photocatalytic degradation of the RhB dye followed a pseudo-first-order reaction according to the Langmuir–Hinshelwood mechanism [30,31], applying Equation (1):

(1) $$\ln C=\ln C_{0}-\mathrm{kt}$$

where C_0_ and C are the initial concentration and concentration at a particular time, respectively, t is irradiation time, and k is the photocatalytic degradation rate constant. The calculated photocatalytic rate constants of Ag/Cu_2_S nanosheets, undecorated Cu_2_S nanosheets, and Cu_2_S nanowires (our previous work) were found to be 0.242, 0.029, and 0.077, respectively. These findings demonstrate that the Ag/Cu_2_S nanosheets had an enhancement that was 10 times larger than that of the undecorated Cu_2_S nanosheets.

**Figure 4:**
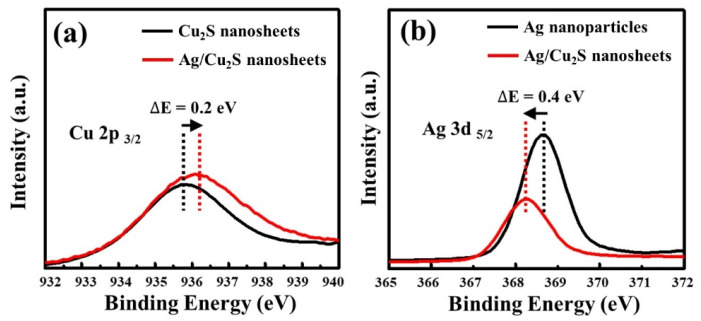
High resolution *XPS* spectra of Cu_2_S, Ag NPs, and Ag/Cu_2_S nanosheets: (**a**) Cu 2p_2/3_ and (**b**) Ag 3d_5/2_.

The additional Ag NP decoration process could decrease the band gap of Cu_2_S nanosheets, and thus favor electron transfers from the valence band to the conduction band. This might lead to an increase in the formation rate of oxidative species, such as the hydroxyl radicals (•OH), as compared to that of the Cu_2_S under the same experimental conditions. Therefore, in order to get insight into the electronic state in the Ag/Cu_2_S nanosheets, XPS analysis was carried out and the results are shown in Figure 4. Figure 4a shows the Cu 2p_2/3_ peak of the XPS spectrum of the Ag/Cu_2_S nanosheets that was broader than that of the undecorated Cu_2_S nanosheets, and consequently right-shifted to the higher-energy region by 0.2 eV. Furthermore, the Ag 3d_5/2_ peak of the XPS spectrum of the Ag/Cu_2_S nanosheets was left-shifted toward the lower-energy region by 0.4 eV, as shown in Figure 4b. The contrasting shifts of binding energy provide direct evidence for the electron withdrawal of Ag NPs from the neighboring Cu_2_S nanosheets.

## 4. Conclusions

In summary, we successfully demonstrated a facile, rapid, and practical approach to synthesize large-scale and uniform Cu_2_S nanosheets and Ag-NP-decorated Cu_2_S nanosheets that have had a size-directing functionality in the reaction process. SEM images indicated that Ag NPs were preferentially deposited on the edge of Cu_2_S nanosheets due to the localization of the electrons on the surface. Localized field amplification that occurred during the excitation of surface plasmons of Ag NPs placed on Cu_2_S nanosheets enhanced UV-visible absorption of the incident photons within the Cu_2_S nanosheet region near each Ag NP. XPS spectra also showed that the Ag NPs on Cu_2_S nanosheets induced a binding-energy shift relative to that of the undecorated Cu_2_S nanosheets. Results provided proof for the electron withdrawal of Ag NPs from the Cu_2_S nanosheets. Ag/Cu_2_S nanosheets that were developed as a new technology in this field tended to follow a superlinear function compared with the Cu_2_S nanosheets, which reacted more slowly in photocatalytic activity. We tested different multifunctionality indicators in various experiments, such as the SERS effect and photodegradation process. The incorporation of Ag NPs highly increased the photodegradation efficiency of the RhB dye molecule under UV irradiation during a 12 h period. The efficient interfacial electron transfers from the excited Cu_2_S nanosheets to the Ag NPs would enhance the photodegradation of the RhB dye molecule. Ag/Cu_2_S nanosheets could perform as a promising dual photocatalyst for SERS applications and future photocatalytic materials.

## Data Availability

Not applicable.

## Supplementary Materials

The following are available online at https://www.mdpi.com/article/10.3390/nano11102508/s1, Figure S1: Structural characterizations of undecorated Cu_2_S nanosheets. (a) The top-view SEM image and (b) EDX analysis of Cu_2_S nanosheets. (c) TEM images of Cu_2_S nanosheets. (d) The selected area electron diffraction (SAED) patterns of Cu_2_S nanosheets. Figure S2: SERS spectra show the reliability of Ag/Cu_2_S nanosheets towards 10^−4^ M R6G dye detection.
