# Supplementary material for: Ag Nanoparticle-Decorated Cu2S Nanosheets for Surface Enhanced Raman Spectroscopy Detection and Photocatalytic Applications
Source: Nanomaterials (Basel). 2021 Sep 26;11(10):2508. doi: 10.3390/nano11102508 (PMC8539072; doi:10.3390/nano11102508)
Supplement: Supplementary file 1 [file nanomaterials-11-02508-s001.zip › nanomaterials-1350015-SI.pdf]

Supplementary Materials

Graphene Oxide and Carbon Nanotubes-Based Polyvinylidene Fluoride Membrane for Highly Increased Water Treatment

Jungryeong Chae [1], Taeuk Lim [1], Hao Cheng [1], Jie Hu [1], Sunghoon Kim [2,*] and Wonsuk Jung [1,*]

[1] School of Mechanical Engineering, Chungnam National University, Daejeon 34134, Korea; wndfud486@naver.com (J.C.); taewook9409@g.cnu.ac.kr (T.L.); chenghao@g.cnu.ac.kr (H.C.); h387669019@gmail.com (J.H.)
[2] Department of Electronics Convergence Engineering, Wonkwang University, Iksan 54538, Korea
* Correspondence: kshoon@wku.ac.kr (S.K.); wonsuk81@cnu.ac.kr (W.J.); Tel.: +82-42-821-6647 (W.J.)

1. Material properties of GO, MWCNTs and ClO^- [1]

1.1. The properties of CNTs

CM-130, outer diameter ranged: 10–15 nm, length: 20μm, S_BET:271m^2/g aspect ratio: 2×10^3, purity > 90 wt%, density: 0.05 g/cm^3.

1.2. The properties of GO

V-20, particle size: 18–104 μm (D50: 50 μm), thickness: 1.1–1.3 nm, carbon: 45–55 %, oxygen: 35–45%, hydrogen ≤ 5%, sulfur ≤ 5%, nitrogen ≤ 1%.

1.3. The properties of ClO^-

Ionic radius of Cl^-: 0.18 nm
Ionic radius of O^{2-}: 0.14 nm
Ionic diameter of ClO^-: calculated by Pauling's method: less than 0.64 nm
Average mass of ClO^-: 51.453 Da

2. Materials and reagents of GMP membrane

Graphene oxide (GO, V-20), Multiwalled carbon nanotubes (MWCNTs, CM-130), Polyvinylidene fluoride (PVDF), and *N,N*-dimethylacetamide (DMAC, 99.0%) were purchased from Standard Graphene, Korea, Hanwha Chemical, Korea, Dongguan East Plastic Trade, China, and DAEJUNG, Korea, respectively.

The reagents of filtration performance were sodium hypochlorite solution (NaClO, 9%) and magnesium standard solution (Mg, 1000 ppm), which were purchased from DAEJUNG, Korea. Sulfate ion standard solution (S_4^{2-}, 1000 ppm) was purchased from KANTO, Japan. Dust powder (ISO 12103-1, A2 fine test dust) was purchased from Powder Technology, US. The turbidity and chromaticity standard solutions were manufactured and used based on drinking water management act criteria methods. Hydrazine sulfate (98%) and hexamethylene tetramine (99%) were purchased from KANTO, Japan. Potassium hexachloroplatinate (IV, 99%) and cobalt(II) chloride hexahydrate (99%) were purchased from Wako, Japan. Hydrochloric acid (35%) was purchased from JUNSEI, Japan. The inflow water concentration of all solutions was adjusted with DI water.

3. GMP Filter Module Manufacturing Process

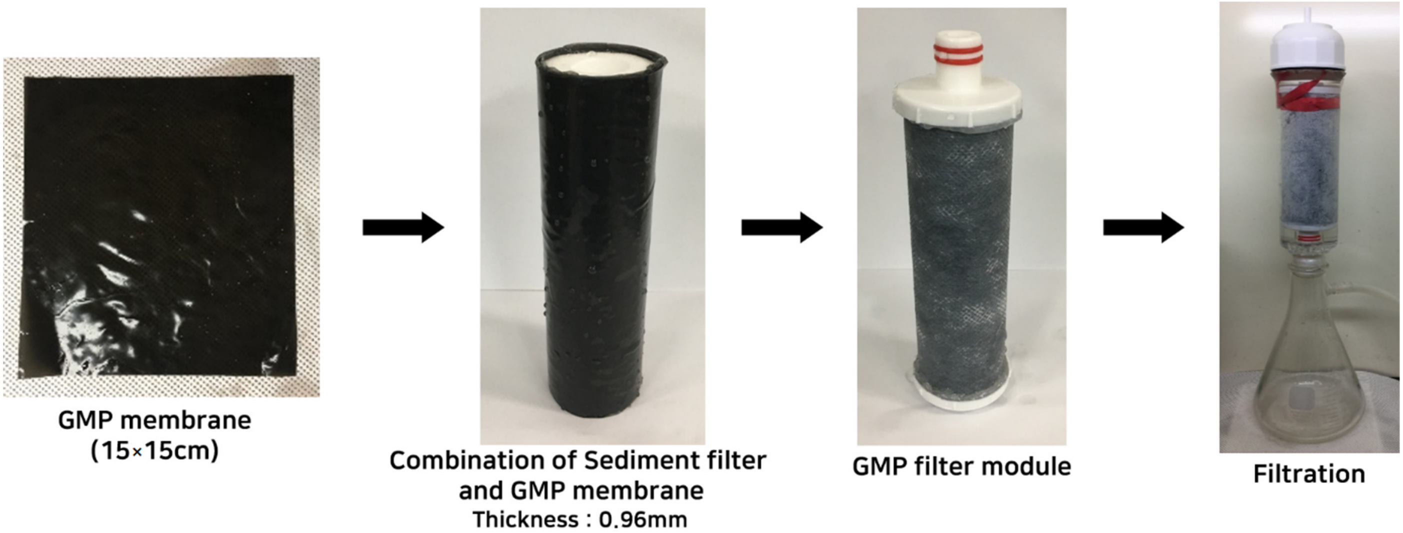

Figure S1. GMP Filter Module Manufacturing Process.

4. Ratio of Carbon Materials of GMP membranes.

Table S1. Ratio of Carbon Materials of GMP membranes at each condition.

Sample name	DMAC (g)	PVDF/DMAC (rw%)	GO/PVDF (rw%)	MWCNTs/PVDF (rw%)
Pure PVDF	100	13		
GO 0.1rw%	100	13	0.1	
MWCNTs 0.2rw%	100	13		0.2
MWCNTs 0.3rw%	100	13		0.3
GO 0.1rw%/MWCNTs 0.2rw%	100	13	0.1	0.2
GO 0.1rw%/MWCNTs 0.2rw%	100	13	0.1	0.3

relative weight of A/B (rw%) = weight of A / weight of B * 100 (%)

5. TEM images of GO / MWCNTs membranes

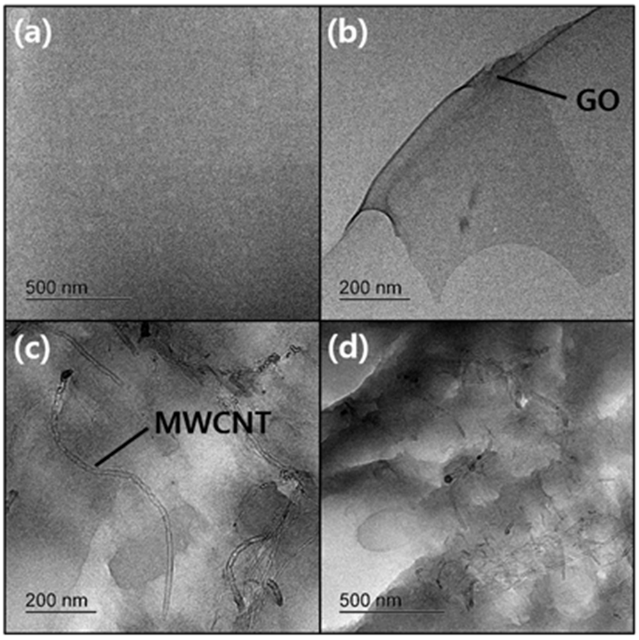

Figure S2. TEM images of (a) pure PVDF and (b)–(d) GMP membranes.

TEM measurements were performed to analyze the structure of GO and MWCNT. We analyze the characteristics of GO and MWCNT membrane through TEM images of pure PVDF membrane and membrane with additional GO 0.1wt%/MWCNT 0.3wt%. Analyzing the measurement results, neither CNT nor GO was found in Pure PVDF. However, GO and MWCNT were observed to be distributed on the membrane surface for GO 0.1wt%/MWCNT 0.3wt% samples.

6. Characterization of Water flux of GMP filter

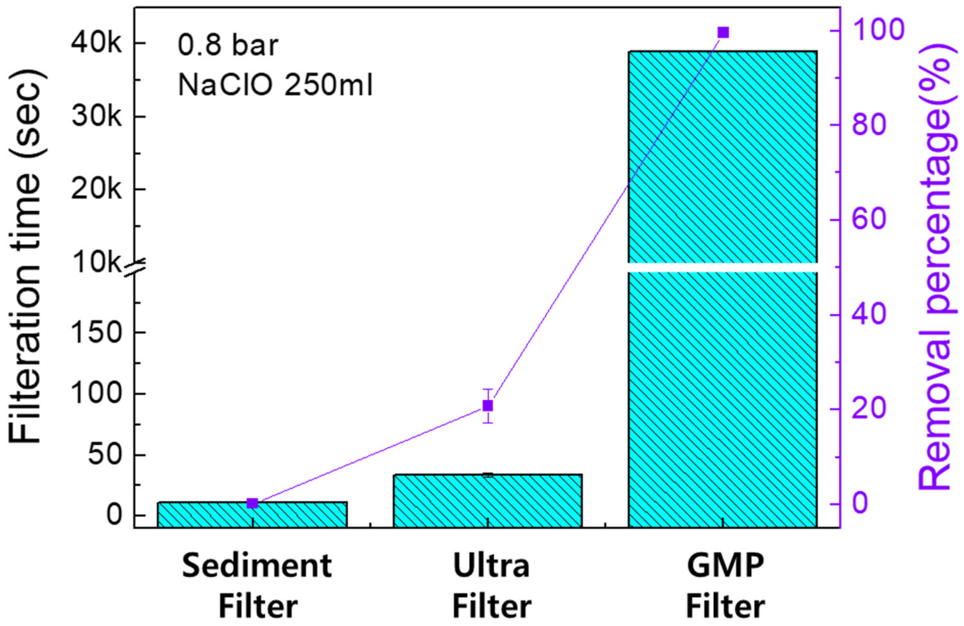

Figure S3. Comparison of filtration time and removal percentages of NaClO, for commercial water filter and GMP filter module, respectively.

7. Analysis of Detection of Graphene Oxide and Carbon Nanotubes in the elution

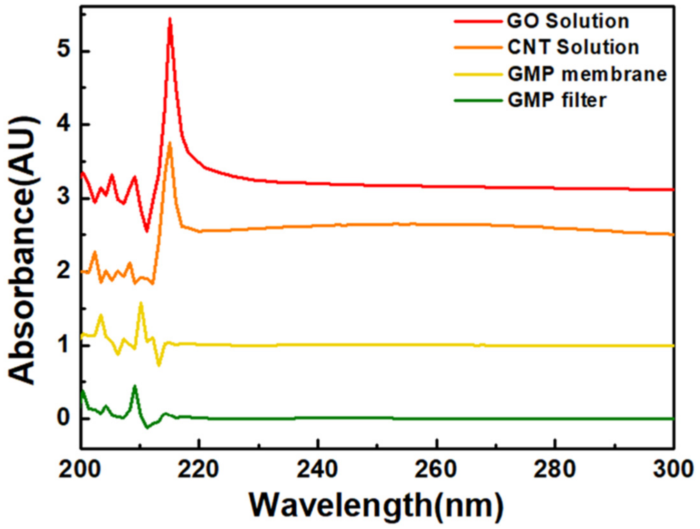

Figure S4. Analysis of detection of graphene oxide and carbon nanotubes in the each condition.

8. Reasons for selection of 6 assessment criteria

In order to evaluate the performance of GMP filters, filtering performance was verified for free residual chlorine, turbidity, and chromaticity, respectively, which are essential criteria of Drinking Water Quality Control Act in South Korea. In addition, the filtering level of ionic material and particle items was analyzed to verify the performance of the separation film. Particle filtration criteria were referenced to US criteria, and ionic material filtration criteria were verified using inorganic (magnesium) and organic (sulfate ion) materials.

References

1. Shannon R. D. Revised effective ionic radii and systematic studies of interatomic distances in halides and chalcogenides. *Acta Cryst*. **1976**, A32, 751-767.
